# Of the Drug Curative

**Published:** 1890-03

**Authors:** P. P. Wells


### OF THE DRUG CURATIVE.

Dr. P. P. Wells.

Communicated by C. Carleton Smith, M. D., Philadelphia, Pa.

(Concluded from p. 63.)

The second case was that of a little girl, five years old, who was suffering from double pleuro-pneumonia of exceeding severity. When the sticking pain in her side was so sharp as all but to prevent her breathing at all, and as to reduce this to the shortest time compatible with life, a teaspoonful of water was offered her, in which a few medicated globules had been dissolved, with the assurance that if she swallowed the water it would relieve her pain. At the very instant the spoon touched her tongue, she shrieked, and declared it did not, but made her worse. It was with the suddenness of thought. There was, however, but this one stab, and it was never repeated. The writer gave the dose. The patient was his own child. The occurrence was before many witnesses. The case, from beginning to end, was of a kind to exclude all possible doubt as to any one of the facts here stated. It is given as a representative of a class which bears important testimony as to the nature of that power which relieves pain and cures disease. That testimony declares plainly, it is submitted, that this power is a *force* and not *matter*.

We have now, at some length, given what we wished to say on the essential nature of disease, its causes, and its cure. As might reasonably have been expected, *a-priori*, in this respect, the three exhibit a beautiful harmony with each other. The relation of cause, effect, and cure being established, and the arrangement of relationship being admitted to the work of an intelligent and disposing will, no other result could have been possible. We think the course of reasoning and illustration presented establishes the fact beyond reasonable cavil that each of these, in its essential nature, is a force, and that, so far as the two last are concerned, their only material connection is that of association, in no way essential to their special action in the living organism as cause or cure of disease.

It is not sufficient to have established these facts to the satisfaction of candid and intelligent minds, as a matter of natural harmony, interesting to observe and contemplate. We are bound to go further, and answer the practical question—What then? What is the practical bearing of the dynamic nature, here proved, on the duties of the physician?

For, if not important in its relation to these, the whole subject is one of curiosity merely, and not worth the trouble of argument or demonstration.

It was for the purpose of presenting its practical relations that the subject has been so far pursued.

The first and most obvious practical inference, from the facts presented, is this: If disease be essentially dynamic in its nature, a change in the state of the living forces, merely, if it be but a force acting in a destructive direction, which, in its normal state, was active for conservatism, then the whole practical duty of the physician in accomplishing its cure is in this one act—to change the *state* of the vital forces to their original conservative balance. If this be the nature of disease, and this the duty of the physician, it is submitted, as obvious on the face of the subject, and to the most superficial observation that these considerations have most important bearings as to the means to be employed for the accomplishment of this end. If this be the nature of disease and its cure, then it is evident there must be a special relationship between the state to be changed and the means by which it is to be restored. And it is further apparent that this relationship must be permanent and subject to no change. It must be a *law* existing in the very nature of the state and the means. And, further, that the cure must be effected through this relationship, and only by means which comply with all its legal requirements. It follows, then, giving of massive doses of drugs to the sick, in quantities great as can be borne without poisonous results, and those selected *with reference to no law* other than that of the caprice of the prescriber, is a crime against science as well as the rights and welfare of the trusting sick. The second obvious inference is that it cannot necessarily require massive quantities of *matter*, merely to change the state of a *force*, even if it be matter which, in its natural state, has associated with it that form of the curative principle which the law of relationship requires for the accomplishment of this change.

It is clear that it can only require so much *force* related to this changed state by the law as may be needed for its restoration.

We have already seen that this does not necessarily bear any proportion to any quantity of matter whatever, but involves only force which, when developed by expansion, as taught in the *Organon*, has parted from its original material association and exists *only as force*, which admits of no measure of its capacity to cure by the application of those rules applicable to the measurements of material forces as applied to other relations. So that it follows not only is the law of selection of the curative dominated by the principles we have advocated, but the form and quantity of the related agent required for the cure as well. The third inference is that so to act on mere *force* as to change its state, only a *mere force* would be likely to be requisite. This would be the conclusion of every philosophical observer, and this is precisely the divine arrangement for the cure of the sick. It has been demonstrated thousands of times in the successful experience of Hahnemann and his followers, their success being ever proportioned to their adherence to this arrangement, as embodied in the laws inculcated.

The fourth practical conclusion is that those who directed their attention chiefly to the material results of the action of this changed force—the proper objects of the science of pathological anatomy—and regarded these as the disease, overlooked entirely the existence of the *cause* in their absorbing contemplation of the *effect*, and by this mistake laid the foundation of all that material philosophy which has pervaded medical science, and from which has sprung all those gross ideas of remedial means and their excessive administration, which has put the art of curing into that bad harmony with disease itself which exhibits both in their true character as destroyers of health and men. Fifth, that those forms of disease which present themselves in apparent changes in the structure of organs and which have been generally, by common consent, handed over to the province of surgery, were, in their origin, like other diseases only a changed force of which those material changes are the product, and that in the onset they were amenable to the curative action of those forces related to their state by the law. And, further, that the great majority of these cases are still, notwithstanding the material change in the structure of organs susceptible of cure by the proper application of the same forces on the well-known principle that the effect ceases when the cause is removed. It is well established in the experience of many who have recognized this fact that the long list of formidable operations which have heretofore characterized the special existence of surgery as a distinct branch of the healing art, may be materially diminished, with no detriment to the interests of society but great gain in the sufferings and mutilations escaped.

Sixth. On this same principle, many diseases heretofore regarded as incurable, and therefore abandoned as without hope, are found to be quite easily removed by going back to the original change in the vital force in which they took their initiative, and adapting a curative force to it, according to the demands of the law of cure discovered. It is no detriment to this statement that the exact nature of this original change is often difficult to discover, or that it is often found in the antecedents of the progenitors of the present sufferer, until it is established in other sciences than that of healing, that difficulty in the practical application of a truth converts it into a falsehood.

Seventh. The dynamic nature of the curative element in drugs explains its successful application in the treatment of mental diseases. These are obviously immaterial. The success which has attended their treatment by the potentized immaterial medicinal powers is, of itself, proof that these diseases are, also, in their nature, in harmony with general diseases and their curative agents. These successes clearly declare that these mental afflictions are but a branch of the general family of diseases, and, like all the other branches are themselves but a changed state of the vital forces.

Eighth. If the above inferences and considerations are well drawn from the premises of this paper, we may add to them this other maxim, in conclusion, that all truth has uses in the practical affairs of life. There is no exception. Least of all are exceptions found in its application to that science of healing which has been heretofore so largely mixed with the false. It is conclusive evidence of practical imbecility when one replies to the presentation of a truth—"*It is of no use!*"

Lastly. It is affirmed of the truths discussed in this paper—the dynamic nature of disease, its cause, and its cure—that they are of the greatest possible use, in this, that they pervade and dominate all practical efforts for healing the sick, made in accordance with the laws appointed by the Almighty for the guidance and control of such acts. To ignore their existence, and yet attempt practical healing, is to wander in a voluntarily chosen darkness when a wise and beneficent Creator has furnished the clear light of a noon-day sun.

